# Boxing as an Intervention in Mental Health: A Scoping Review

**DOI:** 10.1177/15598276221124095

**Published:** 2022-09-09

**Authors:** Johny Bozdarov, Brett D. M. Jones, Zafiris J. Daskalakis, M. Ishrat Husain

**Affiliations:** Department of Psychiatry, Faculty of Medicine, 12366 University of Toronto, Toronto, ON, Canada; 7978 Centre for Addiction and Mental Health(CAMH), Toronto, ON, Canada; Department of Psychiatry, 12220 University of California San Diego, La Jolla, CA, USA

**Keywords:** boxing, boxercise, mental health, anxiety, depression, mixed martial arts

## Abstract

**Introduction**: Physical activity has been shown to have a multitude of mental health benefits. However, there is limited evidence on the specific mental health benefits of boxing. We conducted a scoping review of academic and grey literature to map research of boxing exercises as an intervention in mental health and to identify gaps in knowledge. **Methods**: The authors utilized the PRISMA-ScR methodological approach and guidelines from the Joanna Briggs Institute and a structured search was completed from inception until August 08, 2022. **Results**: We identified 16 documents that used non-contact boxing as an exercise intervention that improved various mental health difficulties. Non-contact boxing exercises, usually in a high-intensity-interval training group setting, provided significant reduction in symptoms of anxiety, depression, PTSD and negative symptoms of schizophrenia. Non-contact boxing provided a cathartic release of anger and stress, with evidence of improved mood, self-esteem, confidence, concentration, metabolic burden, strength and coordination. **Conclusions**: Preliminary evidence indicates that non-contact boxing exercises are a promising intervention to improve mental health burden. Further well designed randomized controlled trials using group, non-contact boxing exercises as an intervention for common mental disorders are warranted to confirm its benefits for mental health.

‘Non-contact boxing appears to provide a cathartic release of anger, aggression, stress, and the dissipation of anxious energy’.

## Introduction

Optimal treatment of mental health symptoms utilizes a bio-psycho-social model of illness; however, in practice, treatment focusses heavily on medication and therapy. Utilizing physical activity is particularly relevant as there are many patients who prefer alternative and non-pharmacological management due to the side effect burden of psychiatric medication.^1^ Studies have shown that physical activity has protective qualities, as well as anxiolytic effects, against emergence of anxiety regardless of demographics.^2,3^ Similarly, there is evidence demonstrating that physical activity can reduce depressive symptoms, and improve mood, sleep and quality of life.^3-6^ Exercises that are more mindful in nature, for example, a mindful focus on deep breathing, have reported better outcomes for mental health than those that are not.^7-9^ In addition, aerobic exercises incorporating high-intensity-interval training (HIIT), have a significant improvement in metabolic and mental health.^8-10^

One form of exercise that uniquely incorporates HIIT with mindfulness (i.e. body awareness and deep breathing) is boxing, which can lead to notable improvements in stress reduction, weight loss, self-esteem, mood and concentration.^11^ In boxing, ‘rounds’ are used to organize a timeline within a HIIT circuit that often includes 2–3 min of exercise followed by 30–60 seconds of rest/recovery. Traditional non-contact exercise techniques used in boxing include stretching, skipping, shadowboxing, pad work, speed bag and heavy bag work. During the rest period, a focus on deep breathing is used for recovery. A cross-sectional analysis of exercise and mental health in 1.2 million Americans from 2011, 2013 and 2015 illustrated a large association of decreased mental health burden in those that engaged in boxing exercises (20.1% lower than those that did not exercise).^7^ Despite the possible benefits of boxing as an aerobic exercise that uniquely integrates HITT and mindfulness, there are no existing systematic or scoping reviews available about the use of boxing exercises as an alternative or complementary intervention to help those with mental health difficulties.

Thus, the objective of this scoping review was to establish a foundational understanding of the use of boxing as an exercise intervention in mental health and examine and identify any existing gaps in knowledge.

## Methods

We used the scoping review methodology as per Arksey and O’Malley with additions from Levac et al. 2010.^12,13^ As per Munn et al (2018), a scoping review methodology was most appropriate for this topic given the scarcity of publications available as seen from our preliminary search.^14^ The review adhered to the recommended scoping review guidelines by the Joanna Briggs Institute^15^ and the Preferred Reporting Items for Systematic Review and Meta-Analysis (PRISMA) extension for scoping reviews (PRISMA-ScR).^16^ Relevant areas of the PRISMA Protocols (PRISMA-P) guidelines were used to facilitate the protocol.^17^ The protocol was published on August 16, 2021 on Open Science Framework which includes template for charting details (https://osf.io/5ztfk).

### Identifying the Research Question

The objective of the scoping review was to identify gaps in existing literature regarding knowledge of relevant studies on boxing exercise interventions for mental health problems for all age groups. The goal was to (1) present an overview of the impact of boxing exercises across mental health symptomatology; and (2) survey the role of mindfulness and catharsis of these exercise interventions.

### Study Eligibility

Studies were included if they used any boxing intervention with at least one related mental health outcome. We defined boxing interventions as having boxing hand work combined with a component of aerobic exercise. This includes interventions described as non-contact boxing, therapeutic boxing, boxercise or group boxing class, as an intervention. Martial arts, Tai Chi or Tai Chi related interventions were not considered boxing interventions by our definition. There was no date cut off criteria. We also critically appraised the quality of identified articles using the Critical Appraisal Skills Programme (CASP) checklist for inclusion.

### Search Strategy & Study Selection

The search strategy was developed in consultation with a medical Librarian (SB) at The Centre for Addiction and Mental Health (CAMH). JB performed the final structured search of Google Scholar, MEDLINE® and PsychInfo. Keywords used included ‘boxing’ or ‘boxercise’ or ‘boxing intervention’ and ‘mental health’ or ‘anxiety’ and ‘depression’ until August 17, 2021 (see online supplementation through Open Science Framework for search). The search was re-run on August 08, 2022. There were no restrictions placed. Additional studies were identified using the grey literature, searching the references lists of included studies.

JB organized the title and abstracts and reviewed publications. Two reviewers (JB and BDMJ) independently scanned titles and abstracts. A two-stage screening process (JB and BDMJ) reviewed the full text, established eligibility and charted the dated. Disagreements were discussed and brought to IH to make the final decision.

### Charting & Synthesis of Data

Charting the data was used to collate all necessary information by JB. As per the protocol, a standardized charting form was developed by the research team that included domains such as article details, study details and design and details of boxing intervention. The charting data was organized into a table (Table 1).

**Table 1.** Characteristics of resources using boxing interventions and mental health outcomes.

Study	Study Design(CASP Scoring)	Population/Purpose	Sample Size	Gender	Mean Age (SD)	Data Collection	Boxing Intervention	Exercise Length	Main Results
(Mackay & Neill, 2010)^34^	Quasi-experimental (+)	Australian, general population near university Use of ‘green exercises’ to reduce anxiety	11	10F 1M	24.36 (2.01)	Pre and post STAI	Instructor led (Group) Boxercise (details unclear)	60 min, once	- Significant reduction of Anxiety
(van Ingen, 2011)^22^	Qualitative (+)	Toronto, Canada Free, trauma- informed, non-contact boxing program for women who have experienced violence	78	78F	16–58	Observational encounters, focus group, interviews	Shape Your Life Program Instructor led (Group) Boxercise-style classes - Warm-up. Rounds on the heavy bag + technical	Unclear length 1x week	- Safe emotional outlet and release of stress and anger
(Hefferon et al., 2013)^11^	Qualitative (+++)	England, referred from Community Mental Health teams Majority with Depression and Anxiety Use of boxing to aid those with mental health experiences	10	6M 4F	36	Pre- and Post-: Intervention Focus Group	Instructor led (Group) Boxercise - Cardio (machine, skipping) - Skills (bag work, pad work) - Strength (resistance exercises)	120 min 1x week for 6 weeks	The class was a catalyst for ‘realigning their lives’ - Social development - Improved concentration and physical health - Less ruminating - Reduced stress - Sense of purpose/community
(Oertel-Knöchel et al., 2014)^20^	Randomized Controlled Trial (+++)	Germany, inpatient (Mixed population with SCZ and MDD) Aerobic exercise to treat the inpatient population. The aerobic exercise used was boxing	8	4M 4F	36.6 (12.9)	Pre- and post- BDI-II STAI PANSS	Instructor led (Group) Boxing exercise - Warm-up (10 min) - Boxing circuit (25 min) - Cool down (10 min)	45 min 3x week For 3 weeks	- Statistically significant reduction in anxiety in MDD, with reduction in anxiety in SCZ - Statistically significant reduction in depressive symptoms in MDD - Statistically significant reduction in negative symptoms of SCZ
(Shultz et al., 2014)^29^	Qualitative (+)	New Zealand obese adolescent boys of Maori and Pacific Island descent Boxing to aid in weight loss and psychological wellbeing	3	3M	14.4 (.2)	Participants and two parents completed semi-structured interviews	Instructor led (Group) Boxing exercise (30 min) - Shadow boxing, bag work, pad work - Progressive resistance training (30 min)	60 min 3x week for 12 weeks	- Psychological benefits that included less aggressive, calmer, more confident and better performance in school
(Cheema et al., 2015)^10^	Randomized Control Trial (feasibility study) (+)	Australian, general population aged 18+ with (BMI) >25 kg/m2 Feasibility and effectiveness of a 12-week boxing training (HIIT) intervention compared with an equivalent dose of brisk walking in obese adults	6	3M 3F	43 (19)	BMI HRQoL	Instructor led (Group) Boxing/HIIT - Warm-up (5 min) - 2 rounds (2 min on, 1 min rest) each of skipping, footwork drills, heavy bag, focus mitts, circular body bag	50 min 4x week for 12 weeks	- Reduced body fat percentage (−13.2%) - Significant improvement in the Physical Functioning, General Health and Vitality domains of HRQoL
Yli-Piipari et al., 2018)^28^	Case series study with pre- and post-test analyses (++)	United States Overweight Hispanic children and adolescents using the CORE (boxing program) to improve metabolic and mental health and motivation	20	16M 4F	11.73 (1.39)	Behavioural Regulation in Exercise Questionnaire Cardiometabolic	CORE non-profit Program instructor led (Group) - Warm-up - Stretches - Cardio and body weight exercises with HIIT boxing exercises - Details unclear	60 min 1x week for 12 weeks	- Significant improvement in intrinsic motivation and amotivation - Improved BMI, and fasting glucose
(Gallenberg, 2019)^25^	Case series study with pre- and post-test analyses (++)	United States Large, public Midwest University listserv To engage men (who had adherence to socialized masculine norms) in an intervention that they would perceive as non-threatening to their masculinity to access psychological services	24	24M	30.5 (10.1)	CMNI-46 ATSPPHS- SF BSI-18	Instructor led (Individual) - 6 rounds (3 min exercise with 1.5 min rest) - Shadow boxing (1 round) - Padwork (3 rounds) - Bagwork (2 rounds)	50 min (20 min tutorial 30 min exercise) 6 sessions, alternating days	- Statistically significant decrease in mental health distress and psychological symptoms regardless of masculine norms - Boxing is a masculine congruent healthy behaviour that improves mental health
(Ghaffar et al., 2019)^26^	Case series study with pre- and post-test analyses (++)	United States Using the Rock Steady Boxing Program to test the effect of non-contact boxing on non-motor symptoms in patients with Parkinson’s disease	44	33M 11F	71	PHQ9 PDQ-39	Rock Steady Boxing Program Instructor led (Group) Non-contact boxing - Warm-up, ringwork, padwork - Bagwork circuit - Core work + cool down	75 min 1x week for 12 to 36 weeks	- Statistically significant improvement in depressive symptoms - Improved Parkinson’s symptoms
(Morton et al., 2019)^21^	Qualitative (++)	Ireland Community based integrated fitness and education substance use rehabilitation programme	17	10F 7M	37	Semi-structured interview	Boxing Clever Program Instructor led (Group) - Strength and conditioning - Fitness training - Pad work - Shadow boxing - Self-defence skills - Technical skills such as stance, blocking and sequences of punches	Unclear length Every other week for 20 weeks	- All reported improved mood - 94% continued abstinence/reduced substance use - Opportunity for self-growth and awareness - ‘Boxing’ identity helped with ‘addict’ identity - Gender inclusive
(Woodhead et al., 2019)^31^	Qualitative (+)	Camden (London), England Free community based mix of non-contact boxing skills with mentoring with life skills *Teachers identified youth who are at risk for social exclusion or crime	8	Unclear	13-18	Observational interviews teacher observation Rosenberg’s Self-Esteem survey Modified Aggression Scale SWEMWBS	Action Youth Boxing Intervention (AYBI) Instructor led (Group) - Non- contact group boxing training & mentoring - 30 min boxing activity - Blend with various activities such as yoga, and nutrition - Relaxation and breathing techniques as part of cool down	60 min 1x week for 3 weeks	- Release of stress an anger - Less aggressive - Improved confidence, self-esteem, empathy and social skills - Improved engagement in class lessons and socially
(Lyon et al., 2020)^23^	Mixed- Method pilot Study (+)	Melbourne, Australia Left/Write//Hook (LWH) - Non-contact boxing + creative writing workshop to aid women who have experienced childhood sexual abuse and trauma find a connection to their body, mind and spirit *Program moved online after week 2 with a focus on virtual boxing exercises	8	8F	27–54	PCL-5 MHC-SF DASS-21	LWH Program Instructor led (Group) - Boxing techniques - Partner work - Bag work - Cardio - Strength-based training skills	120 min (1h writing & 1 h boxing) 1x week for 8 weeks	- Significant reduction in PTSD symptoms (clinically meaningful) - Significant improvement in overall well-being - Reduction in symptoms of depression. Anxiety, stress (not statistically significant)
(Empire Fighting Chance, 2021)^33^	Qualitative (+)	Bristol England Ongoing free community boxing program since 2006 UK’s largest non-contact boxing programme to children at risk of exclusion from school *Adjusted virtually for covid-19	4822	Unclear	8–25	Survey CORE-10	Empire Fighting Chance Program Instructor led (Group) - Warm-up, shadowboxing, technique, bag work, pad work + Mentoring	Unclear length 1x week for 20 weeks	88% reduced psychological distress 87% are more confident 83% have improved self-esteem 70% are happier - Improvements in education, relationships, motivation, aspirations and mental health
(Hermanns et al., 2021)^27^	Mixed-method study with pre- and post-test analyses (+)	United States Using the Rock Steady Boxing Program for Parkinson’s disease to elucidate patients and caregiver’s perception of the non-contact boxing program *Issues related to covid-19 were discussed	6	4M 2F	59–81 and older	CES-D Scale PDQ-39	Rock Steady Boxing Program Instructor led (Group) Non-contact boxing - Warm-up, ringwork, padwork - Bagwork circuit	60 min 2x week for 12 weeks	- Improved mood, reduced depressive symptoms and improved quality of life - Caregivers reported improvement in ADLs and reduced perceived stigma
(Xu et al., 2021)^30^	Feasibility study (+)	China, University students. Use of non–immersive virtual reality (iVR) boxing-inspired exergame, FitXR to improve mental health and reduce stress	15	8M 7F	19.1 (.96)	BAI BDI-II Perceived Stress Scale	FitXR (boxing-inspired iVR fitness game) Individual - 11 different game levels - Punching objects virtually using (jabs, crosses and uppercuts) paired with defence (covering up) and squats to avoid objects	30 min 2x week for 6 weeks	- Statistically significant reduction in depressive symptoms - Slight reduction in anxiety and stress (not significant)
(Gammage et. al., 2022)^24^	Case series study with pre- and post-test analyses (++)	Toronto, Canada Woman-identifying survivors of violence Use of a free, trauma-informed program that reconfigures the sport of boxing into a non-contact form	56	56F	35	Short-Form-WHOQOL Rosenberg Self-Esteem Scale Resilience Scale Personal Agency Scale Physical Self-Efficacy Scale	Shape Your Life Program Instructor led (Group) - Trauma-informed coaching tailored to each individual when possible - Boxercise-style classes - Warm-up. Rounds on the heavy bag + technical	90 min 1x week for 14 weeks	- Statistically significant improvement in physical and mental health, in quality of life and in self-esteem - Significant difference in resilience - Significant improvement in personal agency and perceived physical ability

Abbreviations: SCZ, Schizophrenia; MDD, Major Depressive Disorder; PHQ9, Patient Health Questionnaire (for depression); PDQ-39, The Parkinson’s Disease Questionnaire; STAI, State-Trait Anxiety Inventory; BDI-II, Beck Depression Inventory- II; BAI, Beck Anxiety Inventory; PANSS, Positive And Negative Symptom Scale; HRQoL, Health-Related Quality of Life; BMI, Body Mass Index; HIIT, High-Intensity, Interval Training; SWEMWBS, Shortened Warwick and Edinburgh Mental Wellbeing Scale; CMNI-46, Conformity to Masculine Norms Inventory-46; ATSPPHS- SF, Attitudes Toward Seeking Professional Psychological Help Scale-Short Form; BSI-18, The Brief Symptom Inventory 18; PCL-5, PTSD Checklist for DSM-5; MHC-SF, Mental Health Continuum Short Form; DASS-21, 21-item Depression, Anxiety and Stress Scale; CES-D, Centre for Epidemiologic Studies Depression Scale; PDQ-39, Parkinson Disease Questionnaire 39; WHOQOL, World Health Organization Quality of Life measure. CASP scoring: high quality (+++), acceptable (++) or low (+).

### Quality Assessment

The primary author (JB) utilized the CASP checklist as a guide to further assess the quality of the identified literature. Given that the included articles in this review had mixed study designs, two critical appraisal tools were used, the CASP Qualitative Checklist^18^ and the CASP Randomized Control Trial Checklist.^19^ The CASP Qualitative Checklist was also used for the remaining studies to appraise the literature in terms of validity, results and clinical relevance. Although CASP does not provide a scoring tally, each study was assigned an overall level of evidence as high quality (+++), acceptable (++) or low (+) according to the criteria included in the checklist. The results of the CASP assessment scoring are included in Table 1.

## Results

### Selection of Search Results

**Figure 1.**
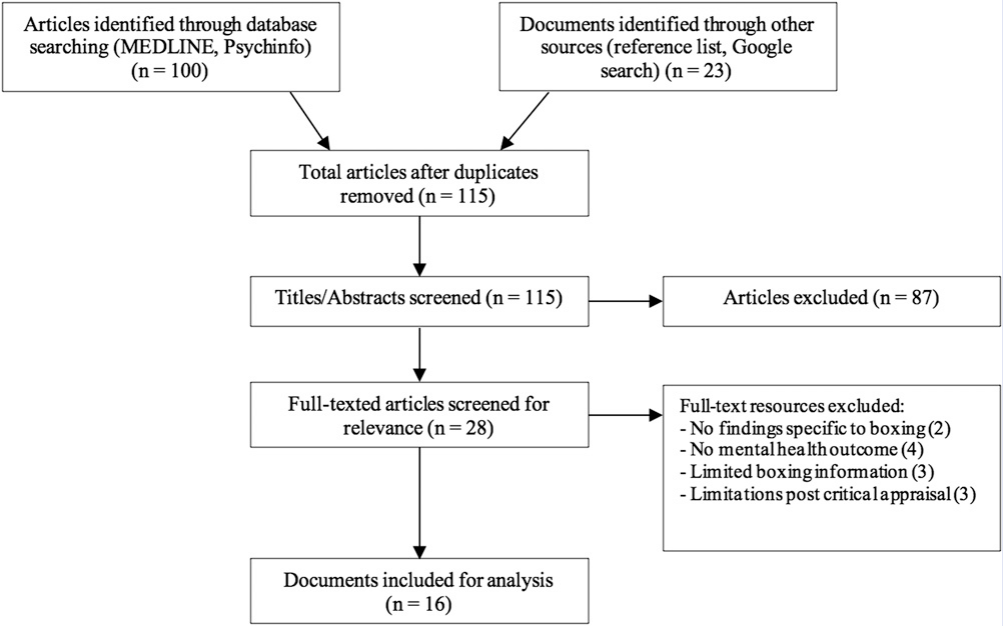
PRISMA flow chart.

A total of 100 studies were identified through database searches. An additional 23 resources were identified through reference list and Google searches. A total of 6 articles were removed as duplicates and the 115 remaining article title/abstracts were screened and 28 studies were read. A total of 16 studies met the pre-determined inclusion criteria following the use of the CASP checklist (Figure 1). Twelve studies were excluded as there was insufficient information related to boxing (2), the boxing intervention (3), a mental health outcome (4) or had insufficient evidence post critical appraisal (3).

### Quality Appraisal

The results of the CASP assessment scoring indicated that the majority of the sources were low quality (56%) with limitations in research design, discussion around bias, ethics and data analysis. Five of the 16 articles were deemed to be adequate (31%) and only two were rated as high-quality papers (12%). The high-quality papers^11,20^ provided a valid study design, comprehensive methodology, results and an unbiased critical discussion of the impact of the results.

### Characteristics of Sources

The majority of studies included were journal articles (81%), while the remainder consisted of reports (12%) and a dissertation (6%). Most were from United States (25%) and England (19%), followed by Australia (19%), Canada (13%), Germany (6%), Ireland (6%), China (6%) and New Zealand (6%). Studies represented a mix with the majority being qualitative (38%) and case series study with pre- and post-test analyses (25%), with fewer being randomized controlled (13%), mixed-method (13%), feasibility (6%) and quasi-experimental (6%). All documents were published within the past 12 years. Majority of the articles studied an adult population (69%) with a few using youth (19%) and two with seniors with Parkinson’s Disease (13%). The study population varied from general population, to those with mental health difficulties,^11^ to participants with substance use disorder,^21^ women who’ve experienced trauma,^22-24^ males conformed to masculine norms,^25^ inpatients treatment resistant schizophrenia or MDD,^20^ Parkinson’s Disease,^26,27^ obese youth,^28,29^ university students^30^ and marginalized youth.^31^

### Variety in Distribution of Boxing Exercises

The majority of studies facilitated the non-contact boxing exercise intervention in a group setting (88%) while two studies completed the boxing exercise individually (13%). All boxing exercise interventions were completed in-person at a physical location except for one paper (6%) which studied an exercise game (i.e. exergame) using a non-immersive virtual reality (iVR) boxing game that incorporated boxing punches and movements.^30^ In over half of the studies, non-contact boxing was supplemented with the additional use of either mentoring, cognitive function drills, mindfulness, meditation, nutritional knowledge, psychological strategies (i.e. journaling, cognitive behaviour therapy techniques) (56%). Non-contact boxing was the main focus of intervention with no obvious supplementation in 44% of the articles.

### Variety in Implementation of Boxing Exercises

The majority of the studies used boxing exercises as an intervention on a consistent weekly basis (94%) while only 6% used it on a one-time basis. The structured implementation of these boxing interventions varied in length from 30 min to 240 min per exercise session with a weekly intervention range from 1–4x per week for 2–20 weeks. The majority (69%) of studies had a range from 45 to 60 min of boxing intervention length. The majority of studies utilized a typical non-contact boxing exercise template for their intervention (94%). This includes beginning with time dedicated to a warm-up and technical work (i.e. shadowboxing or pad work), with the majority of the time dedicated to punching the heavy bag followed by a cool-down period (i.e. stretches, resistance or deep breathing). Often rounds were used to organize the timeline in a HIIT format (i.e. 2–3 min of exercise and 1 min break).

### Importance of Leadership Experience and Community

The majority of boxing classes (88%) were conducted in a group setting and some participants discussed that group classes provided a sense of community and connectedness which made the experience less isolating given their already perceived notion of isolation from their mental illness.^11,32^ Having an experienced boxing coach made participants feel safe and cared for while simultaneously increasing their confidence and self-agency.^11,22-25,33^ There were a few multifaceted, larger community programs that provided psychosocial support in addition to boxing such as aide with transportation, nutritional education, food, psychological strategies, career advice and mentorship.^22-24,26,31,33^

### Mental Health Benefits of Non-contact Boxing Intervention

As it relates to mental health, the majority of articles collectively included results that boxing reduced stress, and improved mood, self-esteem and quality of life (94%). Studies showed significant improvement in overall mood,^20,23,24,26,27,30,33^ reduced substance use,^21^ improvement in self-esteem and confidence,^11,23,24,27,29,31,33^ perceived physical ability,^24^ performance in school,^29,31^ and overall wellbeing and mental health.^10,11,23,24,26,27,29^ With the use of measurement-based care, a few articles reported a statistically significant reduction in specific symptoms burden post boxing intervention. These included symptoms of depression as per PHQ9,^26^ BDI-II^20,30^ and CES-D;^27^ symptoms of anxiety as per STAI;^20,34^ negative symptoms of schizophrenia as per PANSS;^20^ and symptoms of PTSD as per PCL-5.^23^ In addition, there was a statistically significant decrease in mental health distress and psychological symptoms as per BSi-18^25^ and improvement in quality of life as per PDQ-39,^27^ WHOQOL^24^ and HRQoL.^10^

### Boxing as an Outlet for Anger & Aggression

Many studies illustrated the use of boxing (mainly punching a boxing bag) as an outlet to express their anger in a healthy way and to control their overall anger or aggression.^11,21,22,25,29,31^ One participant with a trauma history described ‘whilst anger can fuel a training session, it quickly dissipates and the interplay between the physical and intellectual begins’.^23^ Participants in another study commented that ‘boxing is a way to get anger out’ and ‘I’m angry but instead of me hurting myself, which I always do, I’m learning to channel that outward, like hitting the bags ...every time I come here, it’s always better’.^22^ Boxing appeared to also help release tension, stress, control anger and reduce aggression in youth which helped with productivity in school.^29,31^

### Trauma-Informed Boxing Intervention & Gender Norms

Three studies discussed using a free trauma-informed, recreational, non-contact boxing program for marginalized self-identified women who’ve experienced violence/trauma.^22-24^ Participants felt included in a historically masculine dominated sport and empowered in having a safe/healthy emotional outlet to release their energy/anger. With discussions around anger being an appropriate response to trauma and the use of a ‘movement based program that deploys the fists as weapons and the body as a source of strength and power’ compared to traditional ‘talk therapy’.^22^ Additionally, one participant said ‘By the time we’ve completed the hour of boxing I’m exhausted, but the sadness, the trauma, feels so much farther away than it did at the start’. One article illustrated that boxing intervention appears to also be a healthy help-seeking behaviour for men who reported higher endorsement of masculinity and thus could be an avenue to reach men with mental health burden.^25^

### Elements of Mindfulness in Boxing

A few studies discussed how boxing is a mindful sport given the constant focus on deep breathing, and the mind/body connective awareness needed to stay focused on technical skills while punching a bag/pads.^11,21^ This element of focus led to a reported escape from rumination/emotions.^11,22,23^ Participants wanted a class that was both active and dynamic to leave little ‘idle time’ which prevented excessive rumination.^11^ One articles utilized mindfulness/meditative techniques such as deep breathing as a way to aid in ‘cooling down’ following an intense exercise.^31^

### Perceived Barriers and Adverse Events

Majority of the perceived barriers were socioeconomically related which included transportation, clothing, resources and nutritional snacks which many programs helped to provide.^22,24,31,33^ Two adverse events in terms of musculoskeletal injuries in patients engaged in a boxercise program were noted and they continued with adjusted protocol.^10^ Notably, the troubled youth participants did not use their boxing techniques for violence outside of the gym, such as at school.^31^

## Limitations

Limitations of this review include the use of a non-systematic search strategy and the limited peer-reviewed studies on boxing as an intervention in mental health. In addition, the studies seem to have a high risk of bias, from very small sample size and no justification for sample size, uncontrolled design, lack of clarity about the intervention and fidelity, intervention retention and outcomes that were not validated or appropriate for the purpose used. Questions about acceptability and feasibility of respective interventions for participants, especially with poor mental health, were not summarized in any critical way to inform the future design of research. As indicated by use of the CASP checklist, the majority of the sources identified were rated as low-quality level of evidence.

## Discussion

This review examined boxing as an intervention in mental health. Results are explored further with broader literature with an emphasis on salient points that illustrates the uniqueness of non-contact boxing exercise benefits for mental health.

### Non-Contact Boxing Exercises

Boxing appears unique compared to other HIIT aerobic exercises due to the constant focus on technique, using force to strike an object and interplay of mind/body connection. Many boxing gyms around the world use elements of non-sparring boxing training to create their own HIIT group workout class (similar to those identified in Table 1) with various names such as Boxercise™, ‘boxfit’, ‘boxing class’, ‘cardio boxing’, ‘group boxing’ and so on.^35^ Exercise as a mental health intervention may have an optimal treatment duration of 60–90 min, 2–3x per week over a period of between 6–9 weeks.^7,8,36^ This exercise timeframe was consistent with the majority of the boxing programs identified in this review and is suggestive of an appropriate boxing intervention timeframe.

### Mental Health Benefits of Boxing

There is clear evidence from previous literature that physical activity, whether aerobic, anaerobic or flexibility exercises, can improve mental health and play an important role in the treatment of depression and anxiety.^7-9,37^ Though limited by the lack of high-quality studies, the results of this review suggest there may be mental health benefits of non-contact boxing. The act of punching a bag appears to provide a cathartic release that other physical activities may not provide. This seems to allow for the dissipation of energy leading to improvements in anger, stress, mood, anxiety and quality of life. Boxing and Martial Arts share some commonalities in training in terms of awareness of body/stance, footwork, shadowboxing and various striking techniques.^38^ Our findings are consistent with a recent systematic review and meta-analysis on martial arts training and mental health which indicated a significant but small positive effect on wellbeing (*d* = .346, 95% CI = .106 to .585, *I*^2^ = 59.51%) and a medium effect on internalizing mental health (*d* = .620, 95% CI = .006 to 1.23, *I*^2^ = 84.84%).^39^ Other mindfulness based martial art exercises, such as Tai Chi, have also shown to reduce anxiety and depressive symptoms.^40^ The Rock Steady Boxing program that blends therapeutic, non-contact boxing specifically for individuals with Parkinson’s Disease has demonstrated reduction of symptom burden with improvement in social life, balance, confidence, quality of life and mental health.^26,27,41,42^

### Metabolic Benefits of Boxing

There are significant metabolic and cardiovascular risks for those with mental illness which leads to a greater risk of premature mortality.^43^ For example, patients suffering from depression have an increased risk of developing diabetes,^44^ cardiovascular disease^45^ and obesity.^46^ Non-contact boxing programs were able to improve BMI, fasting glucose, resting heart rate and significantly reduce body fat percentage (*P* = .047) and systolic blood pressure (*P* = .026).^10,28^ Similarly, a study demonstrated that using a type of mindful shadowboxing intervention for patients with type-2 diabetes, and comorbid symptoms of depression and anxiety, provided a significant relief of symptom burden (*P* < .001) and a significant reduction in glycated haemoglobin (*P* = .016), compared to the control group.^47^ As such, boxing may be a feasible and effective complementary intervention for patients with mental health disorders (such as depression and anxiety) who also have comorbid metabolic dysfunction. Notwithstanding, a systematic review identified that non-contact boxing interventions provided physical benefits to patients with diverse severe medical conditions in the area of cardiovascular fitness, upper body strength, body composition, coordination, gait independence and gait speed.^48^

### Elements of Mindfulness

The limited results suggest that components of boxing such as the need for the mind/body connection, the focus on targets while punching, the awareness of balance and an emphasis on deep breathing at the end of rounds may inherently create a mindful activity.^23,31^ However, a major limitation is that none of the studies used measurement-based scales to gather objective evidence around mindfulness and boxing. The Mindful Attention and Awareness Scale (MAAS)^49,50^ could be used to assess the chore characteristics of mindfulness developed post-intervention, whereas the State Mindfulness Scale for Physical Activity (SMS-PA),^51^ could be used to assess the state mindfulness of the mind and body following participation in the physical activity itself. Further studies should consider using such scales to evaluate the level of mindfulness in boxing.

### Service User Perspective

Form a service user perspective, patients found sports and group interventions to be socially inclusive, which provided a sense of non-stigmatizing community that allowed for increased compliance and effectiveness in achieving/maintaining recovery.^52^ In addition, the aesthetics of a gym that is different from the ‘traditional’ community fitness gym seemed to decrease anxiety and encourage participation of exercise while having a trusted instructor seemed to also enhance performance.^53^ Results identified in this review suggest that group boxing classes organically achieve these aspects important to service users. This includes providing a sense of community that is socially inclusive, collaborative, safe and aesthetically unique, while integrating coaching to elevate the exercise experience and improved participation.^11,21,22,24,25,33^ Sports interventions historically have been successful in engaging young male service users and improving quality of life.^54,55^ Boxing as an intervention may help provide access to men who have a tendency to not seek help for mental health concerns due to perceived masculine norms.^25^ Notwithstanding, female service users have expressed positive experiences with boxing interventions suggesting that this intervention is suitable for both genders.^22-24^ Exergames may be useful as a part of mental health treatment with potential to increase access and reduce exercise barriers.^56,57^ Exergames relating to boxing have shown preliminary evidence to reduce depressive symptoms in subsyndromal depression in older adults^58^ and university students.^30^

In terms of potential improvement with exercise engagement, music appears to have a role in exercise that enhances enjoyment, significantly improves physical performance and decreases overall perceived exertion.^59^ While having a choice provides evidence of perceived control that encourages exercise motivation, engagement and completion of tasks.^60-62^ To our knowledge, none of the articles commented on whether music was incorporated and only one article^24^ commented on the need for choice/flexibility to maximize the boxing experience for participants with a trauma background. Implementation of choice and music would be something to consider in the future when providing a boxing intervention.

## Conclusion

There is some preliminary evidence that non-contact boxing interventions may have mental health benefits for symptoms of depression, anxiety, PTSD and negative symptoms of schizophrenia. Non-contact boxing appears to provide a cathartic release of anger, aggression, stress, and the dissipation of anxious energy. With evidence of improved mood, self-esteem, happiness, confidence, self-agency, concentration, metabolic burden, strength and coordination. Boxing provides a safe inclusive environment that integrates community, and leadership. There seems to be limited evidence regarding the inherited elements of mindfulness within boxing and future studies should consider evidence-based scales to report on this. Integrating mindfulness into a group boxing intervention may be a novel and alternative approach to helping individuals with mental disorders manage the symptoms of their illness. Further research should look to test boxing as an intervention in high-quality randomized controlled trials.
